# Effect of COVID-19 pandemic on grade inflation in higher education in Turkey

**DOI:** 10.1371/journal.pone.0256688

**Published:** 2021-08-25

**Authors:** Engin Karadag

**Affiliations:** Department of Educational Sciences, Akdeniz University, Antalya, Turkey; Universita degli Studi di Palermo, ITALY

## Abstract

This study analyzes the effects of the COVID-19 pandemic on grade inflation in higher education. Data were collected from five universities in Turkey, including grades of 152,352 students who attended 2,841 courses conducted by 903 instructors before the COVID-19 pandemic and grades of 149,936 students who attended 2,841 courses conducted by 847 instructors during the COVID-19 pandemic. The results of this study demonstrate that the COVID-19 pandemic causes a marginal increase in grades in higher education when the other factors that might explain the differences are controlled. Grade inflation of 9.21% is the highest ever reported in literature. Compared with a year ago, DD and DC grades decreased 55%; FD and FF grades decreased 31%; and the highest-grade AA increased 41% for courses taken during the pandemic. Additionally, classroom population, academic history of the instructor, class level, field, university entrance scores, and course execution and evaluation (grading) forms of course notes are important determinants. This increase can be explained by the effort of instructors who are accustomed to face-to-face settings. When they suddenly switch to distant education, they might try to grade higher to compensate for the unforeseen negative circumstances.

## Introduction

In December 2019, the discovery of a new type of coronavirus (COVID-19)-infected pneumonia causing serious illness and death was reported by the World Health Organization (WHO) in Wuhan, China [1]. Clinical studies state that the symptoms of COVID-19 include fever, cough, shortness of breath, and sometimes, watery diarrhea [2]. By January 2020, the COVID-19 infection took on the proportions of a pandemic that affected more than 160 countries in less than a few weeks. The number of new COVID-19 cases is rising significantly, particularly in Europe and the US. Turkey, which leads in the number of COVID-19 cases in the world, reported its first case on March 11. Various institutions and organizations took some protective measures in the days that followed. Like all other sectors, the education sector was affected in many ways by the COVID-19 pandemic. The government’s actions are aimed at reducing the spread of coronavirus by introducing measures that limit social contact. Many countries have suspended face-to-face lessons and exams and imposed restrictions on the exchange of students within the scope of the Erasmus program [3]. Primarily, since March 16, 2020, all primary, secondary, and higher education institutions, including all preschool education institutions, were halted for 3 weeks [4]. In view of the rapid increase in the number of COVID-19 cases in Turkey and upon coming to the understanding that the pandemic will last longer, the Board of Higher Education decided to conduct the 2019–2020 spring semester through distance learning, open education [5], and digital learning opportunities by March 26, 2020. This situation is also valid for the fall semester of 2020.

Fortunately, several modern tools can be used to overcome the difficulties of distance learning thrust upon us by the COVID-19 pandemic. Using these tools, the content of previously taught face-to-face courses and lessons can be changed easily. However, there are other important tasks related to the learning process, such as measuring autonomous learning, which can still be challenging without the direct supervision of instructors. All these arguments raise the following concerns: ensuring fair grading to accurately measure the progress of students and helping instructors compare students’ results as to whether they differ from those in previous years. On one hand, higher scores than those in previous years may be associated with cheating in online exams or changes in the format of evaluation tools. On the other hand, lower grades can be attributed to autonomous learning as a less effective teaching method [3]. In this context, this study reduces the uncertainty in the evaluation process in higher education during the COVID-19 pandemic. To measure the effect of the COVID-19 pandemic on grade inflation in higher education, the grades given for the courses during the pandemic setting (PS) and those during the pre-pandemic period (PPP) are compared. The data obtained from five universities in Turkey, with 302,288 students attending 5,682 undergraduate courses for two academic semesters for the academic year 2019–2020, present constraints in face-to-face training.

## Background of grade inflation

Students receive grading to indicate their academic success [6]. With inflated grades, the tendency to question the rationale behind the grading increases [7]. In this respect, “*grade inflation*” is a common explanation for rising notes with regard to giving higher grades to equivalent work. Although empirical literature on grade inflation in higher education outside of the US is inadequate, this subject has been widely investigated in the US [8, 9]. In particular, over the past few decades, *note inflation* claims in higher education have increased in US universities, along with extensive evidence documenting the prevalence and seriousness of the phenomenon. It is known that students spend less time studying in grade-inflated classes. In addition, students who received inflated grades in entry-level or prerequisite classes often stated that they felt inadequate in advanced courses and presented the tendency of grade inflation as the reason [10].

An increase in grade inflation in higher education institutions was noted, i.e., the grades of students began to increase from the 1960s. One of the pioneering studies in literature, a comprehensive survey conducted by Kuh and Hu [11], argued that grades were higher in the 1990s than they were in the 1980s, and average grades increased in all higher education institutions. Researchers claimed that this was because more women attended higher education in the 1990s than did women in the 1980s and that it may not be a direct fallout of grade inflation. However, recent studies disprove Kuh and Hu’s statements. Eiszler [12] studied the 20-year data at a state university in central US and found that the percentage of students waiting for A grade tends to increase notably over the years. Similarly, almost half the students in 2017 graduated with honors from universities such as Princeton, University of South Carolina, and others [13]. In a comprehensive study, Rojstaczer [14] found that grades gradually rose from the 1930s and 1940s. However, a sharp increase was detected in the 1960s, which was balanced in the 1970s. The sharp increase was often due to some instructors hesitating to give low grades at the time because men with low scores were sent to fight in the Vietnam War [11]. After the 1970s, according to Seldin’s [15] calculations, every decade, the average grades began to rise again between 0.10 and 0.15 (range of 0–4). Grade inflation does not only apply to mediocre US universities. For example, at Harvard University, the percentage of A grades increased from 22% in 1966 to 46% in 1997 [16], which resulted in 91% of the 2001 graduates being awarded an honors degree [17]. Graduates of the elite “Ivy League” universities received higher grades than did those of other universities in the US [8, 11, 17–19].

Many factors influenced the increase in grades in higher education. For example, Rojstaczer [14] attributed this increase to three factors: (a) student evaluation of classes became mandatory; (b) students became increasingly career-oriented; and (c) learning outstripped family income. Rosovsky and Hartley [9] stated the reasons for rising grades as the higher incentives arising from the draft of the Vietnam War; response to student diversity, new curriculum, or grading policies; responses to student assessments [20–22]; and growing culture of consumption. Some researchers reported that the need to improve the registration of certain undergraduate programs [21, 23–27] also triggered the elevation of grades. Chan et al. [28], Tampieri [29], and de Witte, Geys, and Solondz [30] added that competition between colleges also encourages grade inflation to put students in better jobs. Moreover, the additional resources provided to state institutions may also lead to note inflation. Hernández-Julián [31] showed that grade-dependent scholarships could lead students to search for easier classes to maintain the required grade point average. Bar et al. [32] examined a policy change that provides information on course notes, and this policy change encourages students to switch to higher-grade courses. This indicates that changes increase grades. Again, many researchers have found advanced social and economic appropriateness related to assessing students’ scores to be one of the main factors contributing to the phenomenon of grading inflation [33–36]. High grades given by instructors are grade inflation for some; however, this does not apply to everyone. Kohn [37] believes that higher grades may result from a good educator, better assignments, a more regular course schedule, and more communication with students concerning course requirements [38]. Winzer [39] argues that institutional changes; changes in student demography (e.g., student entitlement mentality, increase in the ratio of female students, and adult students); uncertainty of faculty–student relationship; and curriculum changes cause an increase in grading.

Note inflation affects not only the academy but also the labor market. Grading inflation causes cumulation at the top end of the grading distribution, which makes it difficult for the best students to stand out. Workplaces can rely on grades to help distinguish talented students; however, because of note inflation and compression, they do not have sufficient clarity for their hiring processes. This prevents a company’s potential productivity and reduces students’ incentives to work hard in the hope of being hired by the best companies [40, 41]. This process reduces the reliability of higher education, which is one of the key institutions for inflation, meritocracy, and upward social mobility [36, 42]. Although it is easy to assume that grade inflation is not a common sociological problem area of interest and that it is only an educational research area, the theories of class inflation generally indicate a structural change in the broader society and social institution of higher education [43], which, in turn, forms the micro-level incentive system. The influence of macro-level structural changes on micro-level social interactions has arguably been the crux of sociological research of organizations [44].

In sum, grade inflation refers to the tendency to reduce academic requirements and give students higher grades than they deserve [45]. In addition, grade inflation is a process followed by higher education institutions that reduces the actual value of an A grade over an average grade value [46]. Grade inflation weakens standards, making it difficult to compare grades with knowledge and qualifications [47]. From this perspective, Crumbley et al. [7] described grade inflation in higher education institutions as a “fatal symbiosis.” There is no consensus on the causes and consequences of grade inflation. Regardless of the reason, there are three issues related to the phenomenon of grade inflation in contemporary higher education. The first is related to the fact that the distribution of letter grades by time increases (inflation), and thus, A and B are given more than C, D, or F. The second is about the potential factors affecting the course grade. The third issue is more novel: whether the COVID-19 pandemic will cause grading inflation in US universities of the proportions seen during the Vietnam War. In other words, did the process of compulsory distance education trigger the inflation of grades? This study analyzes these three issues.

## Hypotheses

This study determines the effect of the COVID-19 pandemic on grade inflation (increase) in higher education. This basic goal leads to the first hypothesis of the study, which can be formulated as **H1**: The COVID-19 pandemic caused a significant increase in grades given to students. To verify this hypothesis, the differences between default class availability, instructors’ academic history, class level, fields, and university entry scores affecting instructor grades to students were analyzed. Thus, purely note inflation is calculated. The second hypothesis, **H2**, is that before the COVID-19 pandemic or during the PS, the population of the classroom, academic background of the instructor, level of the class, fields, and university entry scores significantly influence the grades. The last hypothesis, **H3**, is that during the COVID-19 pandemic, the forms of conducting courses (synchronized, asynchronous, and lecture notes sharing) and the forms of evaluation of courses (online exams, homework, and project) affected the course grade. Therefore, this study seeks answers to the following questions:

1. Does the COVID-19 pandemic have any effect on the notes given by instructors?
2. Are the grades given by instructors during the COVID-19 pandemic affected by class population, academic history of the instructor, class level, fields, and university entry scores?
3. What are the effects of the training and evaluation forms of courses in the COVID-19 pandemic?

## Methods

### Research design

This study, carried out to determine the effect of the COVID-19 pandemic on grade inflation (increase) in higher education, was designed within the framework of secondary research, which includes the analysis of the original secondary data. The secondary research method involves the summarization and collation of already existing data to increase the overall effectiveness of research. In secondary research, data reliability is higher than that in primary studies as data are collected first-hand by organizations or businesses.

### Data set

In secondary research, data expressed as secondary are generally derived from five basic structures: (i) government and nongovernment agencies; (ii) public libraries; (iii) educational institutions; (iv) commercial information sources; and (v) data available online. The secondary data used in this study were obtained from educational institutions, namely, universities. The data included bachelor’s degree grades from five universities in Turkey. Two criteria were used to determine the choice of universities.

■ The first criterion is the university entry percentage rankings. In Turkey, students are placed in universities on the basis of their scores in a national exam covering the subjects of Turkish language, mathematics, science, and social sciences. In this study, the average percentage of university admission was calculated by averaging the percentile of the last student who settled in each program. Next, the universities were sorted by percentile and divided into five groups.
■ The second criterion is grading criteria at universities. Student evaluation strategies can be assumed similar among universities. The evaluation scale used to represent student proficiency (i.e., using the grade scale of 0 to 4.0 points) and semantic grade categories (i.e., A as “perfect,” B as “above average,” C as “passed,” etc.) is similar among universities. However, there are differences between universities, particularly in semantic grade categories. For example, most universities use the traditional A–F assessment scale; however, for each letter grade, there is a plus/minus system (such as A+, A-, B+, B-, C+, and so on), or subdivisions of each letter note (e.g., AA, AB, BA, and BB) is used. In some universities, the A–F scale does not have an E grade. These examples are similar to those of the evaluation scale that universities use in Turkey; however, considering how students are evaluated, there are some discrepancies.

This study uses the grading category presented in Table 1, and a university random sample from each group is determined under the first criterion. Then, by contacting the relevant universities, the raw data files that included information on each course in 2018/2019 spring and 2019/2020 spring academic terms (such as the academic history of the instructors, grade level, exam type, and course instruction methods) and grades of all students enrolled in each course were obtained. Later, some of the original data were removed from the analysis dataset because of the limitations detailed as follows:

■ Following the main purpose of this study, some extensive limitations have been identified in the data. The first is that the study includes only compulsory courses, which is because the literature indicates a higher grading tendency for elective courses [10, 48, 49] and the change of elective courses every year, something that prohibits a standardized comparison.
■ The second limitation is related to departments. The study is limited to fields common to all five universities where data were obtained. These fields include social sciences and humanities (literature, education, business, psychology, and history), science (mathematics, physics, and chemistry), engineering (computer, electric-electronics, construction, and industry), health sciences (nutrition and dietetics, nursing, and health management), and fine arts (painting, music, and graphic design).

**Table 1:** Note categorization system.

Value Over 100 Points	Coefficient of 4	Letter Grade
88–100	4.00	AA
81–87	3.50	BA
74–80	3.00	BB
67–73	2.50	CB
60–66	2.00	CC
53–59	1.50	DC
46–52	1.00	DD
35–45	0.50	FD
0–34	0.00	FF

To calculate the average grade in each course, a system of quadratic coefficients and letters was used in Table 1. A 5-point scale ranging from 4 (AA) to 0 (FF) was used for average grades, i.e., an average grade can vary from 4.0 (a course each student takes AA) to 0 (a course each student receives FF).

Finally, data for the 2018–2019 spring academic semester (before the COVID-19 pandemic) includes 152,352 student grades given for 2,841 courses by 903 instructors. For the 2019–2020 spring academic semester (COVID-19 PS), 149,936 student grades given for 2,841 courses by 847 instructors were used to create a sample. The grades in all courses consist of the final grade obtained at the end of the 14-week academic year.

### Data analysis

The literature given in the previous section reveals that grade inflation differs according to class population, academic history of the instructor, class level, fields, and university entry scores. Many factors can affect the average grade given in a particular course. In this study, five potential bias factors were examined: classroom population, academic history of the instructor, class level, fields, and university entry scores. Each factor was individually examined using correlation, ANOVA, and *t*-tests, and the factors associated with the average course grade are included in the final analysis (ANCOVA) as a common variable. Including these factors in the analysis as common variables, when testing the main relationship, meaning comparing the notes given before the pandemic and in the PS, provide data to check the side effects that the main relationship may cause.

## Findings

### Grade inflation

For the 2019 spring semester PPP courses, 60.6% of the students are graded above the traditionally accepted average of 2.5 (CB and above), while in the spring semester of 2020 (PS), 68.5% of the students were graded CB and above. In addition, 18.6% of the PPP students were given DC and DD (conditional success), and 9.7% received FD and FF (failed) grades, while 12.8% of the PS students received DC and DD, and 4.4% received FD and FF grades (see Table 2). The percentage of students given DD and DC grades in the courses in PS decreased by 55% when compared with the PPP; FD and FF percentage decreased by 31%; however, the percentage of students who were given AA grade increased by 41%. The average grade given by instructors in PPP was 2.47 (*SD* = 1.32), while the average grade in PS increased to 2.75 (*SD* = .71). The difference detected is rather high and statistically significant (*t* = 11.70, *p <* .001). Accordingly, a marginal increase of 11.07% was detected in the average grades in the courses given during PS, and the pandemic settings caused high-grade inflation in higher education. Thus, there was a significant increase in average grading between the spring semesters of 2019 and 2020 (only 1 year).

**Table 2:** Distribution of letter grades given to students.

Grade	2018–2019 Spring semester % of students	2019–2020 Spring semester % of students
AA	15.2	21.5
BA	16.9	17.1
BB	15.3	14.6
CB	13.2	15.3
CC	11.1	14.3
DC	10.2	8.7
DD	8.4	4.1
FD	5.5	2.5
FF	4.2	1.9

### Factors affecting grading

#### Class population differences

The total number of students in the course is one of the factors affecting the grades. In less crowded courses, instructors have more opportunities to get to know students, and training tends to be more flexible when they have more knowledge of the student’s efforts. Even having only a closer relationship can make it less likely for the lecturer to give a “bad” grade for the fear of upsetting students [50]. In this context, the correlation coefficient of relations between the strength of the class present and average lecture grade in PPP and PS is examined. The results showed that there was a meaningful negative correlation between PPP average lecture grade and class presence (*r =* -.23, *p <* .001). However, a positive meaningful correlation was found between PS average course grade and the classroom population (*r =* .12, *p <* .001). According to these two results, the fewer the students in the PPP class the higher the average grade, and the more the students in the PS class the higher the average grade.

#### Class level differences

Another potential deviation factor is class level, that is, in the lectures in the first two grades (first and second grades), the number of students is higher because of repetitions and students who cannot attend the upper classes. According to the ANOVA results, there is a significant difference between average lecture notes for class levels in PPP and PS (*F* = 5.15, 10.42 *p <* .001). Senior classes have the highest average grade (PPP *M* = 3.02; PS *M* = 3.24), followed by third-year courses (PPP *M* = 2.66; PS *M* = 2.78). The second- (PPP *M* = 2.19; PS *M* = 2.51) and first-grade courses (PPP *M* = 2.03; PS *M* = 2.58) have the lowest average grades. Moreover, the independent sample *t*-test results found a meaningful difference between the notes given in PPP and PS for each class level (*p <* .001). In PS, the highest-grade inflation is in the courses given as “1. Grade” (27.1%) and the lowest graded courses are the ones given as “3. Grade” courses (4.5%). As a result, class level affects course grade.

#### Differences in educators’ academic degree

Another potential factor affecting course grade is the academic position of the instructor. The courses in Turkish higher education are given by lecturers and faculty members. Most lecturers have a master’s degree for conducting undergraduate level programs. The faculty members are PhD holders and have a hierarchical structure as assistant professors, associate professors, and professors. In the dataset of this study, 42% of the courses are given by instructors who have the highest two academic degrees (associate professors and professors). According to the results of ANOVA, the average grade (*M* = 2.88, *SD* = .89) in the courses given by the instructors with the highest academic degree in PPP (*M* = 2.21, *SD* = 1.01) is significantly higher than the average grade in the courses given by teaching officers (*M* = 2.62, *SD* = .93) and instructors (F = 9.20; *p <* .001). A similar difference is applicable to PS. The average grade (*M* = 3.22, *SD* = .75) in the courses given by instructors with the highest academic degree in PS is significantly higher than the average grades in courses given by assistant professors (*M* = 2.59, *SD* = .86), lecturers (*M* = 2.87, *SD* = .71), and instructors (*F =* 13.65; *p <* .001). In addition, independent sample t-test results revealed a meaningful difference between the grades given in PPP and PS according to the academic degrees of the instructors (*p <* .001). The courses where grade inflation is highest in the PS are in the courses conducted by the “assistant professors” (17.2%); the lowest grades are in the courses conducted by the “lecturers” (9.5%). Accordingly, the academic degree of the instructor affected lecture grade.

#### Differences in university entrance scores

Another potential factor that affects grades is the university entrance exam, which expresses the preliminary qualifications of students. For higher education institutions in Turkey, students are required to take a two-phased national exam (higher education institution examinations), including question sets of Turkish language, mathematics, science (physics, chemistry, and biology), and social sciences (geography, history, and philosophy). In this context, each undergraduate program in the study is coded into three groups, considering the relevant entry year score (high, medium, and low). Next, the differentiation of average course grades based on university entry score levels were examined using ANOVA. The results showed that there was a difference between average lecture grades based on fields before the pandemic and during the PS (*F* = 7.98, 9.93, *p <* .001). In both semesters, the lowest average grade is for the groups wherein the university entry score is “low” (PPP *M* = 2.14; PS *M* = 2.49), and the highest average grade is “high” (PPP *M* = 2.78; PS *M* = 3.05). In addition, the independent sample t-test results showed a meaningful difference between the notes given in PPP and PS for each entry level (*p <* .001). The groups with the highest-grade inflation in PS comprise the courses of students who entered the university entry score as “low,” and grade inflation in these groups is 14.7%. Thus, the degree of admission to a university affected course grades.

#### Field differences

On the basis of the fields, students are expected to have different qualifications, and the difficulty levels of courses they take vary. An example is courses in fields such as engineering or science, where mainly mathematics and science courses are more difficult and require relatively more effort than that required in courses in the field of social sciences. Another potential factor that affects grades in this respect is the student’s field of education. In this context, the differentiation of average course grades based on the fields was analyzed using ANOVA. The results showed a difference between average lecture notes in PPP and PS in the context of fields (*F* = 10.41, 9.47, *p <* .001). In both periods, the lowest grades are in engineering (PPP *M* = 1.98; PS *M* = 2.34) and health sciences (PPP *M* = 2.12; PS *M* = 2.54), and the highest grades are given in social sciences and humanities (PPP *M* = 3.14; PS *M* = 3.32) and arts (PPP *M* = 3.09; PS *M* = 3.14). In science, the average grades are as follows: PPP 2.65; PS 2.91. In addition, independent sample t-test results showed a difference in meaning between the grades given in PPP and PS in each field (*p <* .001). The areas where grade inflation is highest in PS are “health sciences” (19.8%) and “engineering” (18.2%), whereas the area where it is lowest is “fine arts,” with 1.6%. As a result, significantly higher-grade inflation was detected in the PS in engineering and health sciences when compared with other areas. PS is statistically significant in all areas of note inflation, but the effect in the field of fine arts is practically minimal (partial β-square = .03) yet in other areas, it is at a rather high level (*η*>.21). These findings demonstrate that the PS plays a role in the increase of the grades given in the courses in social sciences and humanities, engineering, science, and health sciences.

#### Difference between PPP and PS grades in potential factors checking

The results of the analysis show that five factors are associated with the average course grades: class population, instructor’s academic history, class level, fields, and university entry scores. After controlling these five factors with ANCOVA, a difference in the grades given before the pandemic and during the pandemic setting happens to exist (*F* = 21.41, *p <* .001). Statistically, once the averages are adjusted after controlling the effects of classroom population, educator academic history, class level, fields, and university entry scores, the grades given during the pandemic process are significantly higher than those given in the PPP. In the context of corrected averages, a marginal increase of 9.21% was detected in the average lecture grades in PS, and even when the factors affecting the grades are under control, the pandemic setting for higher education causes a rather high-grade inflation.

#### Differences in pandemic-special applications

The entire world, including Turkey, had to divert from the usual flow of courses in the PS for higher education. In this part of the study, the impact of types of conducting courses and grading forms on the average grade is examined. During the pandemic in Turkey, the courses are conducted in three different ways: synchronized, asynchronous, and lecture notes loading. The results of ANOVA revealed a significant difference between the average course grades based on the types of execution of the course (*F =* 13.83, *p <* .001). The highest average grade is given in courses conducted by sharing “lecture notes” with students (*M* = 3.13). This is followed by “asynchronous” lessons (*M* = 2.71). The lowest average grade is given in “synchronized” courses (*M* = 2.63). Accordingly, the forms of execution of the courses given during pandemic affect the course grade.

In universities where this study collected its data, in the PS, courses were evaluated in three different ways: online exams, homework, and project. ANOVA results showed a difference between the average course grades that is based on the evaluation type of the course in the PS (*F =* 15.18, *p <* .001). The highest average grade is given for courses evaluated with “assignments” (*M* = 3.21). This is followed by courses evaluated with “projects” (*M* = 2.80). The lowest average grade is given in courses evaluated with “online exam” (*M* = 2.42). Accordingly, the evaluation forms of the courses given during the pandemic affect the average course grade.

## Discussion and results

This study investigates grade inflation, a neglected topic in the higher education literature. Additionally, and more importantly, this study covers the lessons carried out during the COVID-19 PS, which has affected the whole world. Therefore, this study is unique to the literature and provides important evidence for the fact that grading is associated with the PS. This study reveals (*i*) the effect of the COVID-19 pandemic on grade inflation in higher education and (*ii*) grades given in the courses in PS and the grades given in PPP. The phenomenon wherein higher grades are given to equivalent work is commonly explained as “inflation” [49]. The results show that the pandemic causes a marginal increase in grading in higher education, even after controlling the effects of other factors that can explain the differences. The grade inflation of 9.21% reported by this study is the highest ever reported in the literature. A series of studies documented the increase in average undergraduate grades in the past half-century. In US/American universities, where the most common analyses on grade inflation are conducted, average grades have increased significantly over 50 years, from about 2.5 in 1960 to 3.1 in 2006 (on a 4-point grading scale) [14, 49]. Again, Rojstaczer and Healy [51, 52] stated an increase of roughly 0.1 (0 to 4) in every decade since the 1960s. Specifically, it is found that from 1960, the grades increased roughly 0.7 on average in private universities and 0.5 in public universities. Similarly, Summary and Weber [53] found that the average grade of 2.6 (GPA) at a university in southeastern Missouri increased to 3.1 in 2004. Similarly, the grades rose from 2.83 to 2.97 between 1993 and 2004 [54]. While Carter and Lara [10] stated a tendency to increase grade distributions in the US’s UC and CSU campuses between 2009 and 2013, they reported a significant increase in GPA in only half of the UC campuses during this time. Although the size of grade inflation varies according to data sources, evidence shows that when evaluated together, grades increase around 0.1 every decade.

Calculating grade inflation only on grade calculations can cause errors. Some potential factors in the context of years lead to an increase in grade. Increased student diversity, new curriculum or grading policies, importance of student assessments, improved quality of education; and improvement in teaching skills of instructors can be examples of these factors [9, 28, 29]. In addition, some researchers do not believe that there is grade inflation. Mostrom and Blumberg [55] stated that the rise of grades does not necessarily mean inflation, and they argue that the situation they call grade improvement may be it. The instructors who describe the requirements of the course, the evaluation lists, and the interest of students in the classroom are more likely to have students who will really learn and therefore receive higher grades. Again, according to Summary and Weber [53], note change is not due to inflation but due to productivity improvements, which naturally increase students’ learning and understanding. However, the analyses conducted showed that after the effects of these factors were checked, the average increase in grades was roughly half the unconditional increase [49].

These basic discussions on grade inflation do not apply much to the results obtained in this study because the answer to the question of whether the “grade inflation” is observed in the study because of students’ effort, increased diligence, higher-quality student recruitment, or “pure” grade hyperbole is quite straightforward. The analysis covers recent grades obtained only 1 year ago and compares the same courses, meaning that the pandemic only changed the method of conduct (distance education methods and not face-to-face), but the content of the course remained the same. From this perspective, it is clear that in the process of the pandemic, student performance is “inflated” without any improvement. The percentage of students given DD and DC grades during the PS decreased by 55% when compared with the corresponding numbers for the year before; the percentage of students given FD and FF decreased by 31%, while the highest-grade AA increased by 41%. According to a recent study by Hernández-Julián and Looney (2016), 24% of the grades were A, 35% B, 27% C, 9% D, and 4% F. In 2001, the proportion of A rose to 38%, while the proportion of D fell to 6%. Based on the results obtained, the increase observed in 19 years was reached in a year because of the PS. Even though some of them are not very conceivable, there are several possible explanations for this situation. For example, the PS may have seriously increased the grades since the instructors cannot devote time for assessments. It can be said that seniors are discriminated positively because of their closing graduation if the pandemic affected their situation somehow. Of course, the first and the most plausible explanation for this increase in the grades given is the instructors’ incompetence related to distance education. Since the Turkish higher education system in face-to-face classroom environments is already problematic, it will not be fair to expect a perfect transition of teaching in virtual and digital environments [4]. The second explanation is as follows: Kebritchi, Lipschuetz, and Santiague [56] detected three key factors affecting the success of online courses in higher education based on 104 research overviews: (*i*) student, (*ii*) content, and (*iii*) instructor factors. If we put aside student-related factors, effective online course content is only possible through the efforts and competencies of the instructors. However, because of the COVID-19 pandemic, the training that was planned and started as face-to-face (late January, early February) transferred back to online, and contents developed for face-to-face education have been moved directly to the online environment in Turkey as well as in the rest of the world. Again, according to the authors, online education takes at least 2–3 times more preparation, etc. when compared with face-to-face training. Despite both these situations as well as the will to conduct objective tests in crowded classrooms, examinations consisting of unequal difficulty distribution must have affected the grade inflation.

The results of the study confirm some key findings in the literature. For example, both in the PS and before the pandemic, the academic background of the instructor, the class level, the fields, and the university entrance scores are substantial determinants of the average course grades. Various studies have shown that student grades are related to the academic degree of the instructor [48, 57–59]. Research on this topic has demonstrated that low-grade faculty members consistently give far higher marks than high-grade faculty members [48, 58, 59]. However, the findings obtained in the study are not compatible with the relevant literature. It was found that in Turkey, the professors and the associate professors gave the highest grades, and the assistant professors gave the lowest grades. There are several possible explanations for this incompatibility. Entrance courses in Turkey are not preferred to be given by professors and associate professors when compared with standard practice in Anglo-Saxon countries, and assistant professors mainly conduct these courses. The findings of this study and results in the literature demonstrate that the lowest grades are given for the introductory courses because the number of students is relatively higher due to retakes and because of the difficulties that junior students face during adaptations. Another possible explanation comes from the cultural background of Turkish higher education. In Turkey, professors in faculties do not devote much time to the assessments [60], which are often carried out by their assistants. Another finding that should be interpreted along with this result is that the lowest marks are given to courses given in the first year. It is also about the higher education culture to give low marks in first-grade courses when compared with other classes. Instructors tend to punish students implicitly for potential problems that may occur in the future.

Based on the fields, there are conflicting results in the literature related to the change of lecture grades. In some studies, students in science-related fields received lower grades when compared with students of non-science fields [45, 52], and some studies reported no difference based on fields [10]. In this study, the results obtained were compatible with those of the research studies in the first group. Both during the pandemic and pre-pandemic periods, the highest grades are in the field of social sciences and humanities and the lowest grades are given in engineering. Engineering courses do have a reputation for having strict requirements, with a significant number of their students failing each year. Consequently, the average grade of these courses is significantly lower. This result obtained is within the expectation of this study. Again, as in previous studies [10, 61], a meaningful and positive relationship is found between university entry scores and good grades. For example, a 20% increase in the median of entry points will cause an increase in the marks given to students by 9%. This effect is marginally higher than the 2.5% and 5% difference reported by both Carter and Lara [10], and Johnes and Soo [61].

As previously stated, during the pandemic the grades are on a marginal hyperbole, and there is very high-grade inflation. Some factors of this marginal increase are identified in the study. The fewer the students the higher the average grade: this applies to the pre-pandemic classes. Yet, during the PS, this case is the opposite. The findings for pre-pandemic grades follow the literature. For the less crowded classes, instructors have the opportunity to get to know students, give more feedback about their homework, and so on. In addition, instructors tend to be more flexible when they have more knowledge of the student’s efforts. Even something as ordinary as having a closer relationship with students can reduce the likelihood of the instructor giving a “bad” grade and risking the student getting upset [10]. However, in the PS, this condition has disappeared. This situation also eliminates midterm evaluations. When all midterm evaluations are coupled under a final evaluation for the entire semester by a homework or project, assessments in crowded classrooms may be made faster and be done without paying attention to student development. Another finding that supports this situation is that the highest average grades are given in the PS in the courses carried out by sharing “lecture notes” with the students. Hence, if the instructors spent as much effort, they wanted their students to spend as much labor. This situation, carried out in the form of sharing lecture grades—the educator has spent a low level of labor—and assessments in which they can allocate less time for each student in the crowded classrooms, works in the student’s favor. In contrast, instructors have given a lower grade in “synchronized” courses, a labor-intensive process.

In summary, with the rapid spread of the COVID-19 pandemic, it has become a necessity for educators all over the world to pursue educational processes on various platforms that are products of modern and advanced technology. Decisions such as isolation, social distancing, and quarantine made by countries unexpectedly and suddenly forced face-to-face education to turn into distance education within days [62]. So much so that all academics in the world literally moved online overnight. Everything in higher education (lecture, exam, meeting, etc.) had to be conducted online in a few days. Overnight, the overall structure of programs and units, plans, academic rules, and processes was shattered. The findings obtained in the study revealed a significant picture that the higher education system could not resist this sudden change. The first reason for this situation is that educators who are used to face-to-face teaching and assessment methods cannot adapt to online education pedagogy. The statement of the UNESCO Director-General that “The region without a map has been entered, that is, the borders are crossed,” referring to distance education, is an indicator of this situation [63]. Again, the report published by the OECD [64] reveals that during the COVID-19 pandemic, educators and administrators of educational institutions have insufficiencies in areas such as distance education, structuring online classes, and supporting students. Both the studies in the literature and the findings of this study, by putting aside the longstanding arguments about whether there would be a future of online teaching and learning in higher education [65], revealed the necessity of redesigning and structuring online teaching and for both educators and students to acquire competencies in online teaching pedagogy. Again, the findings of the study put forth the idea that some universities prefer constructive measures such as “improving the assessment and evaluation skills of instructors, organizing exams in accordance with online education pedagogy.” Some other universities prefer non-constructive measures, such as “putting pressure on instructors to evaluate student performance with lower grades, taking inhuman measures to ensure exam safety (mirrors, multiple cameras, etc.)” to prevent grade inflation in both online and face-to-face education and protect their prestige. In addition, unexpected grade increases trigger the application and structure of the exams to depend on the multiple-choice question format heavily, causing the evaluation of pure knowledge instead of evaluating student skills.

Finally, this study reflects not only an example in Turkey but also points out potential grade inflation caused by the pandemic in other countries. However, the time frame used in this analysis is short, only 1 year, and some important factors in the empirical analysis could not be controlled. These factors include a detailed description of the learning, teaching, and evaluation strategies used in distance education courses, as well as a long period of identification of individual characteristics of the educators. The inclusion of these factors in future empirical analysis will be productive for future research. In the future, it will also be interesting to study this situation in the context of various countries. It will also be interesting to examine this situation in the context of various countries in the future. According to the results of the study, the following recommendations could be made to administrators and policymakers of higher education institutions:

■ The process we went through made certain that distance/online education is a part of higher education. From this point of view, increasing priority should be devoted to distance/online training competencies of educators.
■ Without increasing the existing online education capacities of higher education institutions and online education competencies of educators, grades should be given as successful/unsuccessful in compulsory courses and these courses should not be included in the General Weighted Grade Point Average.
■ Guides directed to quality assessment-evaluation practices in online education should be composed.
■ The lowest grades were assigned in the online exams during the pandemic, and a major cause of this situation is large class sizes in Turkey. Therefore, class sizes should be considered when making the choice of exam type. In particular, the evaluation of the courses with smaller class sizes should be conducted with projects.
